# Genome relationships and LTR-retrotransposon diversity in three cultivated *Capsicum* L. (Solanaceae) species

**DOI:** 10.1186/s12864-020-6618-9

**Published:** 2020-03-17

**Authors:** Rafael de Assis, Viviane Yumi Baba, Leonardo Adabo Cintra, Leandro Simões Azeredo Gonçalves, Rosana Rodrigues, André Luís Laforga Vanzela

**Affiliations:** 1 Laboratório de Citogenética e Diversidade Vegetal, Universidade Estadual de Londrina, 86057-970, Londrina, Paraná Brazil; 2 Departamento de Agronomia, Universidade Estadual de Londrina, 86057-970, Londrina, Paraná Brazil; 3 Laboratório de Melhoramento Genético Vegetal, Universidade Estadual do Norte Fluminense Darcy Ribeiro, Campos dos Goytacazes, Rio de Janeiro, 28013-602 Brazil

**Keywords:** Chili peppers Cytogenomics, FISH, Plant genome, Transposable elements

## Abstract

**Background:**

Plant genomes are rich in repetitive sequences, and transposable elements (TEs) are the most accumulated of them. This mobile fraction can be distinguished as Class I (retrotransposons) and Class II (transposons). Retrotransposons that are transposed using an intermediate RNA and that accumulate in a “copy-and-paste” manner were screened in three genomes of peppers (Solanaceae). The present study aimed to understand the genome relationships among *Capsicum annuum*, *C. chinense,* and *C. baccatum*, based on a comparative analysis of the function, diversity and chromosome distribution of TE lineages in the *Capsicum* karyotypes. Due to the great commercial importance of pepper *in natura*, as a spice or as an ornamental plant, these genomes have been widely sequenced, and all of the assemblies are available in the SolGenomics group. These sequences were used to compare all repetitive fractions from a cytogenomic point of view.

**Results:**

The qualification and quantification of LTR-retrotransposons (LTR-RT) families were contrasted with molecular cytogenetic data, and the results showed a strong genome similarity between *C. annuum* and *C. chinense* as compared to *C. baccatum*. The *Gypsy* superfamily is more abundant than *Copia*, especially for Tekay/Del lineage members, including a high representation in *C. annuum* and *C. chinense*. On the other hand, *C. baccatum* accumulates more Athila/Tat sequences. The FISH results showed retrotransposons differentially scattered along chromosomes, except for CRM lineage sequences, which mainly have a proximal accumulation associated with heterochromatin bands.

**Conclusions:**

The results confirm a close genomic relationship between *C. annuum* and *C. chinense* in comparison to *C. baccatum.* Centromeric GC-rich bands may be associated with the accumulation regions of CRM elements, whereas terminal and subterminal AT- and GC-rich bands do not correspond to the accumulation of the retrotransposons in the three *Capsicum* species tested.

## Background

Plant genomes are composed of repetitive and non-repetitive portions, organized in families according to the nature of their sequences, mobility throughout genomes and localization in the chromosomes [1, 2]. Transposable elements are virus-like sequences, and they are grouped into two classes, namely Class I or retrotransposon and Class II or transposon-like. The retrotransposons are the most common elements in plant genomes [1], and they use polygenic chain enzymes, such as reverse transcriptase, for retrotransposition via an intermediate RNA molecule [3–5]. Transposons, on the other hand, use different enzymes, such as transposase (transposons), helicase/replicase (helitrons), polymerase B (polintons) and tyrosine replicase for cryptons [2, 4], to reposition themselves along the genomes.

The LTR-retrotransposons are classified into *Copia*, *Gypsy,* Bel-Pao, retrovirus, and endogenous retrovirus superfamilies, and they may be differentiated by the protein domain organization on the polygenic sequence [2, 5, 6]. The superfamilies *Copia* and *Gypsy* differ from each other by the location of integrase in the polygenic chain: when the integrase is positioned upstream of the reverse transcriptase the element is recognized as a *Copia* retrotransposon, whereas a downstream position identifies the *Gypsy* members [5, 7]. Both superfamilies are subdivided into many lineages [4, 5], with the *Gypsy* taxon encompassing, for example, clades named of Athila/Tat and chromoviruses with CRM and Del lineages [5, 7]. Similarly, *Copia* retrotransposons are grouped into other lineages, such as the Ivana/Oryco, Tork and others [5, 7]. Because LTR-RTs present independent activity in chromosomes and different fates in genomes, closely related species may exhibit variability in their occurrence, amount and chromosome distribution, which influence “fluctuations” in DNA C-values (DNA amount in a haploid nucleus, in picograms) [8–10].

The presence of TEs within genomes may influence gene expression and function. Depending on their insertion region, TEs may change the transcript splicing/processing and coding regions (see [11]). In plants, the LTR-RT lineages may be clustered in different chromosome regions, regardless of the superfamily to which they belong. This may be instanced in the accumulation of *Copia* elements in the proximal chromosomal regions in many plants [12, 13]. Depending on the *Gypsy* lineage, their position along the chromosomes can be much more diverse. Whereas CRM elements are often localize at centromeric regions [14, 15], Athila/Tat and Del elements were found in heterochromatic and euchromatic regions, often scattered across the chromosomes [14, 16]. However, when the retrotransposons are localized using FISH probes made for superfamilies and not specifically for each *Copia* or *Gypsy* lineage, the FISH signals may appear with a scattered profile, as observed in the chromosomes of *Copaifera* [17]. Studies in samples of Solanaceae have shown that *Gypsy* retrotransposons may be associated with heterochromatin at pericentromeric regions of *Solanum* chromosomes [18]. The accumulation of *Gypsy*-Del elements has been reported in both heterochromatic and euchromatic regions of *Capsicum annuum* [19].

*Capsicum* species are an excellent model to investigate the dynamics and distribution of LTR-RTs, because of their relatively large genomes, grouped within 2*n* = 24 and 26 chromosomes, and DNA C-values range from 3.16 to 5.77 pg, in which the repetitive DNA families may represent more than 70% of the genomes [20]. *Capsicum* species present a great diversity of repetitive DNA families, especially regarding number and distribution of ribosomal sequences and heterochromatin sites [21–27]. In this context, the present study aims to acknowledge and compare the occurrence and distribution of LTR-RTs on the chromosomes, focusing on *Gypsy* superfamily that has been predominant in *Capsicum* genomes, as well as their association with the diversity of other repetitive sequences. Peppers are among the most important vegetables in the world due to their high versatility and wide range of applications in cooking, industry, and decoration [28]. Therefore, large investments have been made to obtain high throughout sequences of *C. annuum*, *C. chinense* and *C. baccatum* [20, 29]. This large data volume has increased the possibility of studying and comparing the genomic organization of these species from the cyto-molecular point of view.

Given the gaps in knowledge regarding the repetitive fractions of *Capsicum* species, some questions about the dynamics and distribution of LTR-RTs remain unanswered, such as: Are the heterochromatin rich regions collocated with TE rich regions? Do closely related genomes share the same LTR-RT families concerning quantity and chromosome localization? Our discussion focused on the characterization of retrotransposons based on a broad cytogenomic comparison using the repetitive fraction available in the large *C. annuum*, *C. chinense,* and *C. baccatum* datasets.

## Results

### Comparative analyses based on the conserved domains of transposable elements

The high coverage sequencing scaffolds of *Capsicum annuum* (3.07Gb)*, C. chinense* (3.22Gb) and *C. baccatum* (3.01Gb) from *Pepper Genome Platform* were used for the analysis. The search based on conserved coding domains of polygenic chain (POL) of retrotransposons showed that fractions related with conserved protein domain of reverse transcriptase, integrase and RNase H represents 2.75, 17.14 and 2.47% in the *C. annuum, C. chinense* and *C. baccatum* datasets, respectively (Tables 1 and S1). When Class I and II elements were compared, conserved sequences of Class I elements were more abundant in the three datasets. The Class I was more accumulated in *C. annuum* and *C. chinense* (> 90%) and less accumulated in *C. baccatum* (~ 70%). The conserved sequences similar to Class II elements were less represented with < 10% in these datasets (Tables 1 and S1).

**Table 1:** Frequency and relative values of repetitive fraction in the datasets of the three *Capsicum* genomes

Species/Lineages		***C. annuum***				***C. chinense***				***C. baccatum***			
		num seq	NR (%)	size bp	size CR%	num seq	NR (%)	size bp	size CR%	num seq	NR (%)	size bp	size CR%
Copia	Sirevirus	7421	1.69	1,339,493	1.59	9176	2.29	1,361,598	0.26	3463	0.94	546,478	0.69
	Osser	45	0.01	1335	0.00	65	0.02	2534	0.00	6	0.00	241	0.00
	Tork	5380	1.22	1,645,586	1.95	2378	0.59	357,575	0.07	8773	2.37	5,231,667	6.58
Gypsy	Chromovirus	321,932	73.28	58,580,214	69.41	278,519	69.43	493,702,847	95.71	78,757	21.28	15,931,607	20.03
	Non-chrom.	15	0.00	935	0.00	37	0.01	1242	0.00	13	0.00	970	0.00
	OTA	70,160	15.97	13,807,983	16.36	74,530	18.58	14,025,454	2.72	226,143	61.11	34,401,828	43.24
Other LTRs		12,836	2.92	5,071,268	6.01	886	0.22	44,305	0.01	3845	1.04	1,439,107	1.81
Non-LTR-RTs		0	0.00	1,866,004	2.21	3305	0.82	296,857	0.06	3648	0.99	1,201,048	1.51
Endogenous Virus		1371	0.31	127,214	0.15	1392	0.35	71,067	0.01	1171	0.32	185,499	0.23
Transposons		30,738	7.00	5,840,435	6.92	28,627	7.14	5,135,873	1.00	29,582	7.99	7,154,489	8.99
5S rDNA		717	0.16	89,820	0.11	1370	0.34	172,715	0.03	199	0.05	30,499	0.04
45S rDNA		1267	0.29	1,086,206	1.29	849	0.21	637,555	0.12	18,075	4.88	14,858,366	18.68

Repetitive fraction comparison in *Capsicum* genomes. Num seq – number of sequences found after the Blast rounds. NR (%) – relative value. Size bp – total length of a repetitive class. Size CR (%) – percentage that the class represents in all the scaffolds

**Fig. 1:**
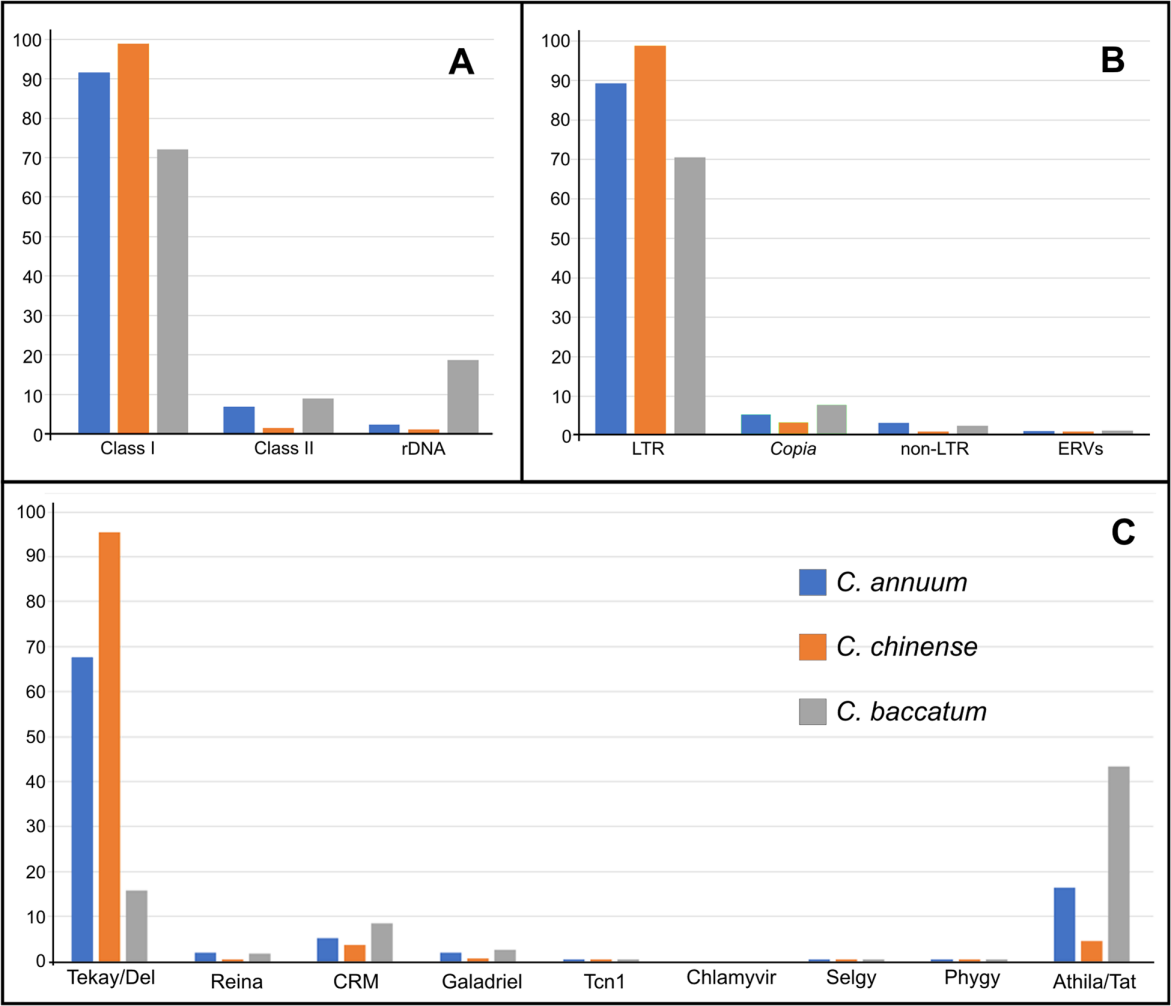
Comparison of the relative distribution (%) of repetitive DNA families among three *Capsicum* datasets. **a** Note that the Class I elements are more common than Class II and rDNA sequences in the three cases. **b** LTR-RT elements predominated over non-LTR and ERVs, but note that the *C. baccatum* genome exhibited 30% fewer sequences than other two datasets. Observe also that *Copia* superfamily elements were less accumulated (< 10%) than *Gypsy* ones. **c** Observe that the accumulated elements were Tekay/Del, Athila/Tat, and CRM, but, except for CRM lineage, there was a big difference in the quantity of the elements in each dataset

After organizing the Class I sequences as LTR, non-LTR and endogenous retrovirus, the percentages of LTR elements showed more similarity between *C. annuum* and *C. chinense* (89.32 and 98.78% respectively) than in *C. baccatum* with 70.55%. Both non-LTR and ERVs sequences were less accumulated, and they were not equally distributed among species: 2.21 and 0.15% in *C. annuum*, 0.06 and 0.01% in *C. chinense,* and 1.51 and 0.23% in *C. baccatum* (Table 1). Sequences recognized as *Copia* superfamily members exhibited low representativeness in all three datasets (< 10%), while *Gypsy* members were the most accumulated. *C. annuum* and *C. chinense* exhibited 85.77 and 98.43% of the *Gypsy*-related sequences respectively, while in *C. baccatum*, they were 63.27% (Table S1). In *Gypsy* superfamily, the Tekay/Del elements were predominant in *C. annuum* and *C. chinense*, with 67.68 and 95.43% respectively, while in *C. baccatum* the Tekay/Del elements presented only 15.7%. The Athila/Tat clade was predominant in *C. baccatum* (43.25%) than in *C. annuum* and *C. chinense* at 16.36 and 2.72%, respectively (Tables 1 and S1). Other *Gypsy* lineages, such as CRM and Galadriel, had lower representation (see Tables 1, S1 and Fig. 1c).

The other elements belonging to the non-LTR groups, transposons, for example, were less accumulated in these three datasets, such as in LINE and SINE (< 5%), ERVs (< 1%), CACTA (< 0.1%), hAT (< 2%), MuDR (< 0.1%) and Helitron (< 0.1%). Nevertheless, Sola elements presented an interesting contrast, representing 5.75 and 7.31% in *C. annuum* and *C. baccatum* datasets respectively, and only 0.94% in *C. chinense* dataset. Ribosomal DNA was also estimated, once literature shows a large variation in the number of rDNA sites among these species. Although 5S rDNA showed no great variation among the three species, the 35S rDNA sequences exhibited a contrasting accumulation, with 18.68% in *C. baccatum,* 1.29% in *C. annuum* and 0.12% in *C. chinense* (Tables 1 and S1).

### *Gypsy* autonomous elements dominated the datasets

The search for putative autonomous retrotransposons was focused on *Gypsy* superfamily members (Tekay/Del, CRM, and Athila/Tat lineages), once their sequences were the most accumulated in the three datasets.

The characterization of retrotransposons was first based on LTR_STRUC [30] output file of *Capsicum annuum* (considered here as “reference”)*,* which resulted in 254 sequences. From these, only four sequences from CRM, three from Tekay/Del and two from Athila/Tat lineages were characterized. Other 267 sequences that have been identified using the BLAST tool came from *C. chinense* and *C. baccatum* datasets, and that were contrasted against *C. annuum* dataset. The BLAST identified four sequences from CRM, and seven from Tekay/Del lineages as putative autonomous elements.

The sequences characterization was based on LTRs’ presence (Dotter l/b [31]), annotation of GAG and polygenic chain (Artemis [32]) and minimal size of elements (> 3.500 base pairs long). The pseudomolecules that were identified as non-autonomous retrotransposons were those that they did not exhibit one or more LTRs, lost genes from the polygenic chain or ORFs, or had large inverted stretches. After two rounds of alignments (ClustalW [33] followed by Mauve [34]) with putative autonomous sequences, they were organized in groups within each lineage. The Tekay/Del sequences were clustered in five groups, with sequences varying from 8105 to 8902 bp length. The sequences of group 2 were shared between *C. annuum* and *C. chinense*, while those of groups 1 and 5 were exclusive for *C. annuum*, and those of groups 3 and 4 were exclusive for *C. baccatum* (Figure S1). The CRM sequences were clustered in three groups, varying from 5259 to 7328 bp in length. Except for groups 1 and 2 that appeared in *C. annuum* and *C. baccatum,* the others were shared among the three species (Figure S2). The two sequences of the Athila/Tat lineage were distinct from each other, making these two groups exclusive of *C. annuum* (Figure S3).

**Fig. 2:**
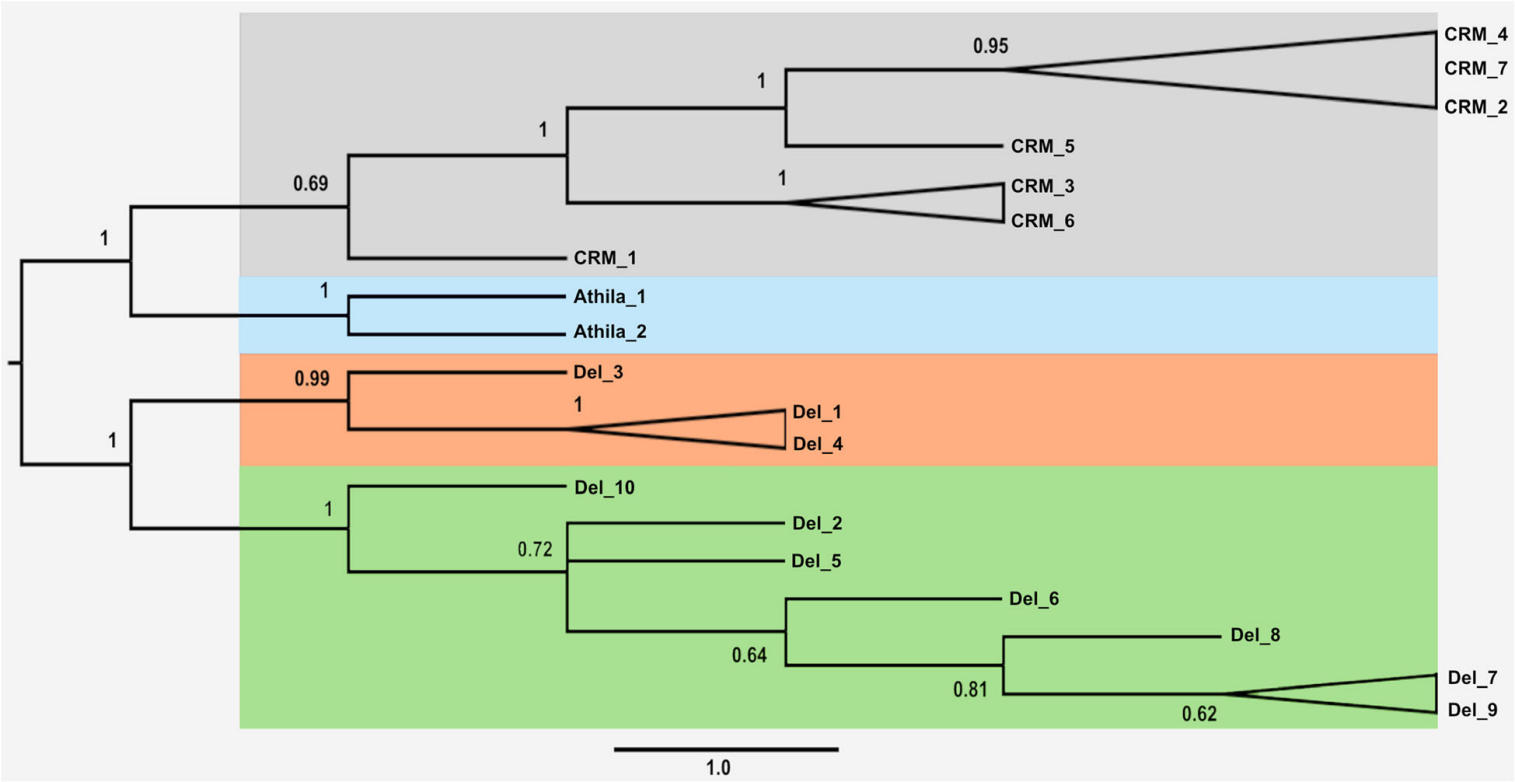
Phylogenetic tree based on putative autonomous sequences of *Capsicum* using the maximum likelihood method with bootstrap 1000. CRM sequences organize four groups (grey, see also the Figure S2) and Athila sequences two groups (blue, see also the Figure S3). The Del sequences were organized in two well-supported groups (orange), corroborating with the MAUVE alignment (Figure S1), and the third group of sequences without well-supported values (green), except for the sequences Del_7 and Del_9 (Figure S1) corroborating with the MAUVE alignment

**Fig. 3:**
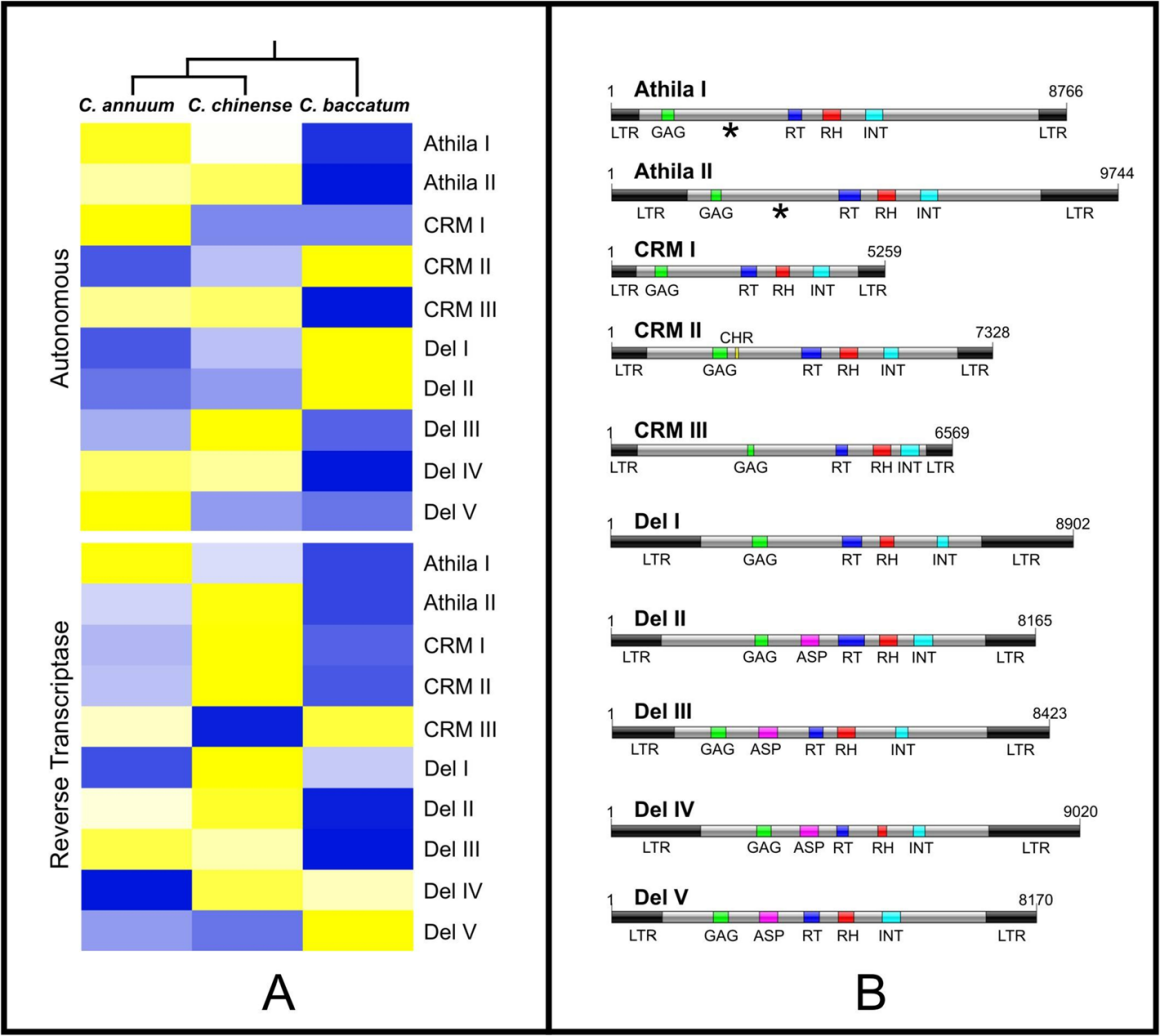
Comparative distribution of the putative autonomous LTR-RTs and the reverse transcriptase sequences along the *C. annuum*, *C. chinense,* and *C.* *baccatum* datasets. **a** The Annuum clade, composed by *C. annuum* and *C. chinense* and the Baccatum clade (*C. baccatum*) can be distinguished by the dendrogram on top of the heatmap. In the heatmap, lower and higher accumulation (blue, intermediate and yellow, respectively) represent the amount of conserved sequences found in each dataset. Image shows that Athila/Tat elements accumulated more in *C. annuum* and *C. chinense* (marked in yellow and light-yellow), the CRM groups were differentially accumulated, highlighting the absence of CRM III and the predominance of Del I and II in *C. baccatum,* and bigger accumulation of Del III, IV, and V in *C. annuum* and *C. chinense*. The reverse transcriptase of these elements exhibits a similar pattern of distribution than the one observed for the complete elements, Athila/Tat I and II were more accumulate in *C. annuum* and *C. chinense*, respectively. CRM I and II were more accumulated in *C. chinense*, while the group CRM III was more accumulated in *C. baccatum*. *Capsicum annuum* and *C. chinense* had a bigger accumulation of Del I, II and II than *C. baccatum*, while the groups Del III and IV exhibited more accumulation in *C. baccatum*. **b** Graphical representation of LTR-RTs groups. LTR – long terminal repeat, GAG – nucleocapsid, RT – reverse transcriptase, RH – RNAse H, INT – integrase, ASP – aspartase. Asterisks present in Athila illustrations refer to the hallmark ORF for this lineage. Note a difference in extension (bp length) among elements, including GAG and POL positioning, and LTR sizes. Note also that only CRM II exhibits a chromodomain sequence and that all the Del elements present an additional aspartase locus

The bootstrapped maximum likelihood phylogenetic tree was performed with complete sequences and organized them in three clades: CRM, Athila, and Del (Fig. 2). The CRM sequences were clustered into four groups: A) CRM_2, CRM_4 and CRM_7; B) CRM_5; C) CRM_3 and CRM_6; and D) CRM_1. The Athila sequences presented two groups within *C. annuum*. The Del elements were more diverse, being organized into three well-supported groups: A) Del_3; B) Del_1 and Del_4; and C) Del_7 and Del_9. The remaining Del sequences did not form well-supported groups (Fig. 2). One sequence was selected from each cluster for a graphical representation (Fig. 3b).

The clusters of putative autonomous elements were evaluated by their accumulation and distribution in each dataset, in order to compare the probable dynamic of these retrotransposons in the genomes differentiation. In panel 3A, the color scale represents how accumulated these elements are in each dataset (blue represents less accumulation and yellow, more accumulation). In general, *C. annuum* and *C. chinense* shared similar sequences in relation to *C. baccatum*, following the phylogeny proposal for the genus. An exception was observed for Del I (Del_1) group, which on the heatmap (Fig. 3a) exhibited greater accumulation in the *C. baccatum* genome. The same was observed for the group Del V (Del_8 and Del_10), which despite being composed of sequences from *C. baccatum,* was more accumulated in *C. annuum*. When the reverse transcriptase regions of these putative elements were analyzed, this tendency, although not the same as in the complete elements, was retained, with some exceptions such as the accumulation of the group Del II, which was composed only of sequences from *C. baccatum* (Del_5, Del_6, Del_7, and Del_9), exhibiting more accumulation in *C. chinense*.

### Comparative cytogenetics

**Fig. 4:**
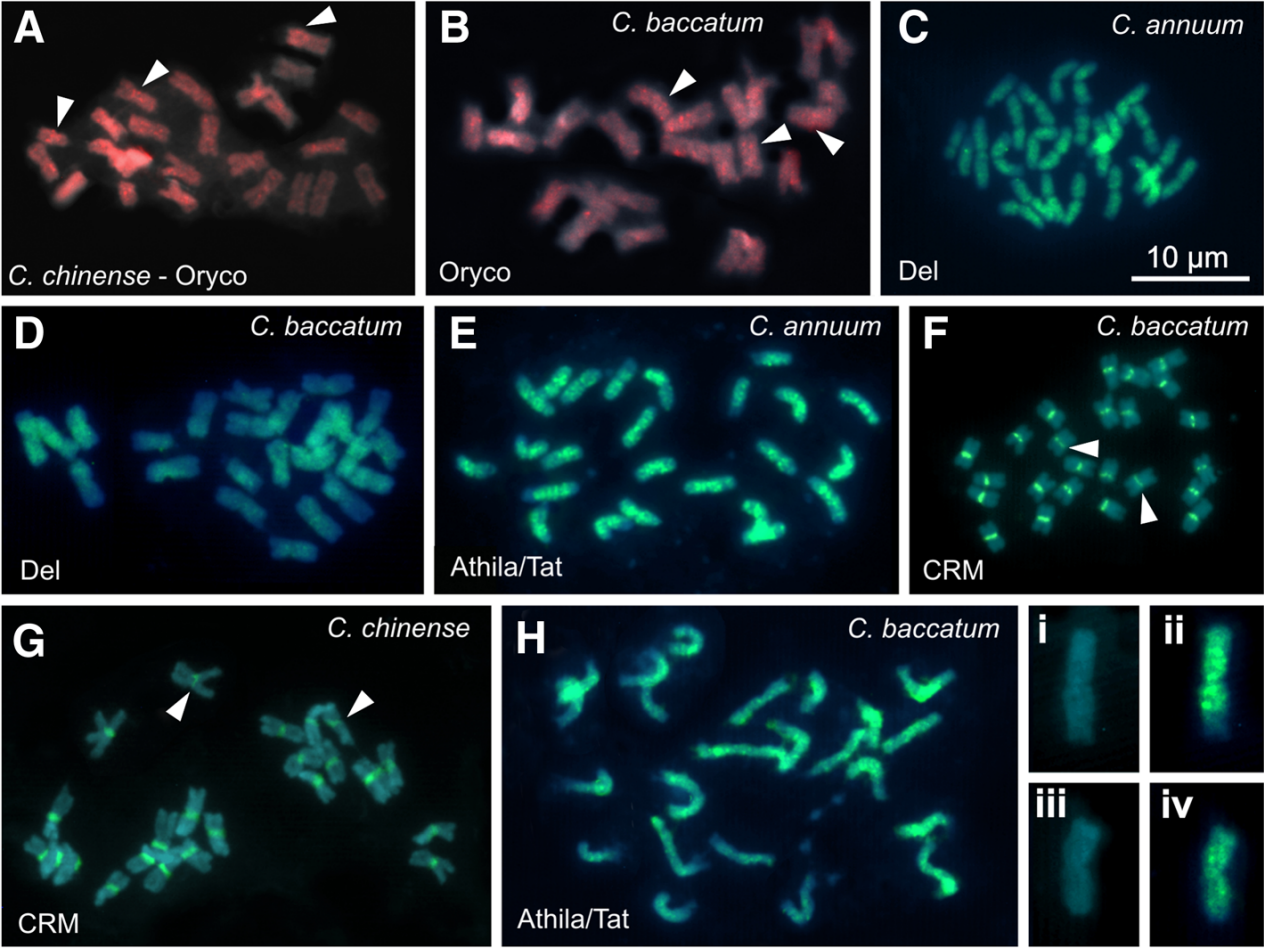
FISH using LTR-RTs probes against metaphases and prometaphases of *Capsicum* species. Chromosomes were counter-stained with DAPI (blue), *Copia* probes with Cy3–11-dUTP (red) and *Gypsy* probes labelled with biotin-11-dUTP / avidin–FITC conjugate (green). The *Copia* Ivana/Oryco probe showed few hybridization signals scattered along chromosomes, with a low accumulated profile in both *C. chinense* (**a**) and *C. baccatum* (**b**). The *Gypsy* Tekay/Del probe exhibited hybridization signals dispersed along the chromosomes in the three species, but with a larger accumulation in *C. annuum* chromosomes (**c**) than the other two species, such as in *C. baccatum* (**d**). The *Gypsy* Athila/Tat probe showed brighter hybridization signals than Tekay/Del, accumulating in the pericentromeric to interstitial regions of all *C. annuum* chromosomes (**e**), differently of *C. baccatum* because some chromosomes accumulated many signals and others very few (**h**). The boxes **i**, **ii**, **iii** and **iv** are highlighting differences in the pericentromeric and interstitial Athila/Tat signals in two *C. baccatum* chromosomes. The *Gypsy* CRM probe showed FISH signals accumulated in the centromeric regions, but with two pairs in each species with much less intense signals. Note the arrows in *C. baccatum* (**f**) and *C. chinense* (**g**). The bar represents 10 μm

The sequences of the reverse transcriptase of the most representative LTR-RT lineages were aligned, and four consensus sequences were used for the primer design: three for the *Gypsy* superfamily (CRM, Tekay/Del, and Athila) and one for the *Copia* superfamily (Ivana/Oryco). The data is presented in Tables 1 and S1. Fluorescence in situ hybridization assays revealed either scattered or clustered signals, depending on the LTR-RT lineage analyzed. The probe for Ivana/Oryco lineage (*Copia*) showed a hybridization profile with a few signals scattered along chromosomes, with clear differences between the pairs and, sometimes, with small interstitial and proximal dots (see arrowheads in Fig. 4a, b and Figure S6a-f).

The Tekay/Del probe showed scattered signals along the chromosomes in three species (Supplementary figure 4). Although the signals observed in *C. annuum* (Fig. 4c) and *C. chinense* (Supplementary figure 4g) were more evident in the interstitial and proximal regions of all the chromosomes, in *C. baccatum*, the signals accumulated in half of the chromosomes and were very weak in the others (Fig. 4d). These results confirm the observations from bioinformatic analysis, which shows a greater accumulation of Tekay/Del sequences in *C. annuum* and *C. chinense* than in *C. baccatum*.

The Athila/Tat probe also exhibited scattered signals along the chromosomes, being more accumulated in the interstitial regions. In *C. annuum*, except for a pair with less intense signals, the remainder exhibited brighter signals at the proximal to interstitial (close to proximal) regions, without any signals in terminal ones (Fig. 4e), similar to those found in *C. chinense* (Supplementary figure 6 g-i). *Capsicum baccatum* exhibited four chromosomes with less intense signals and brighter FISH signals in the remaining chromosomes. However, four chromosomes showed stronger signals than those observed in *C. annuum* and *C. chinense* (Fig. 4h). The CRM probes showed FISH signals accumulated in the proximal regions of all chromosomes in the three species (see Fig. 4 f, g and Supplementary figure 5). Nevertheless, there was a clear difference in signal intensity among chromosome pairs, with a minor signal in a pair of *C. baccatum* and another in *C. chinense* (see arrowheads).

**Fig. 5:**
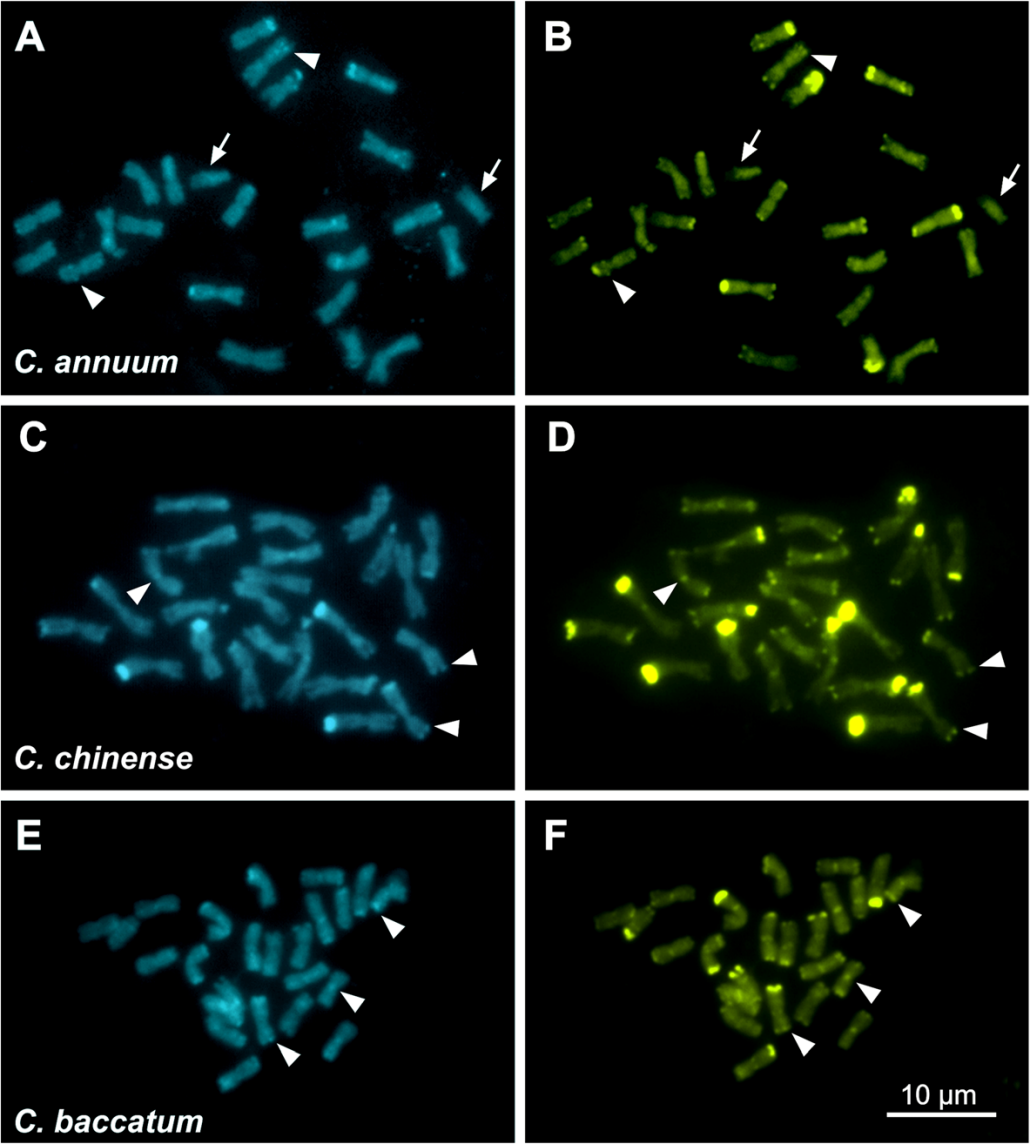
*Capsicum* species show considerable diversity in the C-CMA/DAPI banding profiles. Observe that *C. annuum* presents four chromosomes without fluorescent signals. C-DAPI interstitial dots was detected in three chromosome pairs and terminal bands two pairs (**a**), all of them were co-located with C-CMA bands. Ten pairs exhibit C-CMA signals, being three pairs with stronger terminal and the other as the terminal to subterminal small signals (**b**). *Capsicum chinense* showed four pairs with strongest DAPI signals, besides minor centromeric, interstitial and terminal signals on a few chromosomes (**c**), while C-CMA bands were observed in all the chromosomes, varying as strongest terminal bands in seven pairs, minor centromeric bands in six pairs and as terminal and interstitial dots in ten pairs (**d**). Some of these bands have been evidenced by DAPI and CMA_3_ (**c**, **d**). Observe that *C. baccatum* exhibits six pairs with minor terminal and six with centromeric and/or interstitial C-DAPI (**e**). C-CMA signals were detected in four pairs as strongest terminal bands, but minor terminal bands were observed in all chromosomes, as well as minor centromeric in almost all the chromosomes (**f**). The bar represents 10 μm

The C-CMA/DAPI banding was performed to verify that LTR-RTs’ accumulation areas corresponded to the AT- and GC-rich band regions as well as to check if the diversity in the distribution profiles of repetitive sequences is equivalent in these species. *Capsicum annuum* showed two pairs without bands, three pairs with terminal C-DAPI^+^ dots co-located with more intense C-CMA^+^ bands and terminal C-CMA^+^ bands in 11 chromosomes, including three pairs with stronger terminal C-CMA^+^ bands (Fig. 5a, b). *Capsicum chinense* exhibits a larger number of intense C-CMA^+^ bands (seven pairs), and of these, two pairs exhibited intense adjacent C-DAPI^+^ bands. Smaller terminal C-CMA^+^ bands were observed in 11 chromosomes, and proximal bands were observed as centromeric dots in nine pairs. Some of these bands were evidenced with DAPI and CMA_3_ staining. Thinner interstitial bands were stained with C-DAPI but not with C-CMA (Fig. 5c, d). *Capsicum baccatum* showed six pairs with terminal dots and six with centromeric and/or interstitial C-DAPI bands. In three pairs, these bands appeared as CMA^+^/DAPI^+^. The strongest terminal C-CMA bands were detected in four pairs in addition to thinner terminal bands in at least one arm of all chromosomes. Proximal centromeric dot bands were observed in 11 pairs, of which only three also appeared as DAPI^+^ (Fig. 5e, f).

## Discussion

### Differential accumulation of repetitive DNA families on Capsicum genomes

TEs can move through genomes, representing an evolutionary force that modifies genome structure via mechanisms, such as illegitimate recombination, gene capture, shuffling of regulatory motifs and the generation of new functionality or silencing (see [35]). In the last instance, transposable elements may cause a change in the genomes’ global structure and fluctuations in genome size [10]. They have been useful to compare genomes and karyotypes in evolutionary studies as well as other applicable approaches, such as the study in grapes and blood orange that showed the origin of alterations in the expression of some genes after the insertion of TEs next to them [36].

The ‘Mobilome’ occupies the largest portion of plants’ genomes and play an important role in the physical and functional aspects of chromosomal structures, such as those of the CR lineage (centromeric retrotransposons) that are associated with chromosomal kinetics [15, 37]. In some monocotyledons, for instance, the Mobilome represents around 75% of the genome, such as 80% in maize [6], and LTR-RTs are the most dynamic elements found within the genome [7, 38]. In *Nicotiana attenuata* and *N. obtusifolia*, for example, these elements reach up to 81 and 64% of their respective genomes [39]. The study of LTR-RTs in a chromosomal landscape may assist the understanding of some extent of the regulatory potential of TEs along chromosomes, and may also hold the prospect of its possible application in crop breeding programs, such as peppers. Maize is a good example because several TE families described near the genes have been identified as enhancers or repressors under stressful conditions [40, 41].

Previous studies addressing TEs in the Solanaceae family have shown that the *Gypsy* elements of *Solanum lycopersicum* are more abundant than the *Copia* superfamily members [42], although an approach using only autonomous elements showed a predominance of the Tekay/Del (*Gypsy*) and Tork (*Copia*) lineages in this species [43]. This suggests that estimates may vary when considering only autonomous or include sequences of non-autonomous elements. The present results in *Capsicum* indicate that only 0.002% of the *C. annuum* dataset corresponds to putative autonomous elements, followed by 0.001% in *C. chinense* and 0.004% in *C. baccatum*. The remaining sequences correspond to non-autonomous elements. It is important to highlight that the three genomic datasets were obtained by high-covered sequencing [20, 29], which may support the assembly of the pseudochromosomes as complete elements. Even though the *Capsicum* sequencing does not cover the entire genome (the sequencing part comprises 87% of the *C. annuum*, 94% of *C. chinense*, and 83% of *C. baccatum* genomes) [20, 44] it can be stated that TEs occupy an important fraction of *Capsicum* genomes. The major part of coding repetitive fractions relates to fragments of non-autonomous elements, which may be amplified by the activity of the autonomous [36]. This might explain the high percentage of LTR-RT fragments in these three datasets.

According to Qin et al. [29], the *Gypsy* members were the most abundant LTR retrotransposons in *Capsicum*, with the highest insertion activity among Solanaceae species. When comparing *Gypsy* and *Copia* lineages in this plant group, the percentage of LTR-RTs for *C. annuum, C. chinense,* and *C. baccatum* was 89, 98, and 70%, respectively. This result pointing out a predominance of *Gypsy* (> 70%) over *Copia* superfamily (< 10%). A contrasting accumulation of Tekay/Del, Athila/Tat and CRM lineages of *Gypsy* were noted in these three genomes. These data point toward the importance of LTR-RTs’ fate in the process of genome organization and differential accumulation between related species, even after considering that *C. annuum* and *C. chinense* (Annuum clade) are closer when compared to *C. baccatum* of the Baccatum clade (see supplementary figure S7) [45]. Kim et al. [44] reported that *Solanum* is a closely related genus of *Capsicum* and shares a common ancestor 19.6 MYA. These authors demonstrated *C. annuum* and *C. chinense* share a common ancestor 1.14 MYA, while these species shared 1.74 MYA with *C. baccatum*. These three species also differ in the accumulation of 35S rDNA, with about 20% more sequences in *C. baccatum* than in *C. annuum* and *C. chinense*, besides the number of rDNA sites in the chromosomes [25]. These genomic differences may be responsible for certain difficulties in performing interspecific crosses between these species of distinct clades because of the pre- and post-zygotic barriers, as reported by Manzur et al. [46] and Cremona et al. [47].

The differential activity of retrotransposons among close genomes was also reported in *Helianthus* [48] and *Solanum* [49, 50], and these results corroborate with those obtained in the present study regarding *Capsicum*. The present data have also shown differences among *C. annuum* and *C. chinense*, especially in Tekay/Del, ERVs, and Line-RTE accumulation, suggesting that other elements, besides LTR-RT ones, evolve independently. The differential accumulation of Tnt1 retrotransposons in *Nicotiana* may be a good example to understand and to support the idea of an independent fate of TEs on genome differentiation [51, 52].

### Recovered Del, CRM, and Athila/Tat autonomous elements support the *Gypsy* LTR-RTs’ predominance

The ability of retrotransposons to activate and invade plant genomes may be associated with some internal and external factors, such as biotic and abiotic stresses, breeding processes, injuries, climatic changes, polyploidization, hybridization, and other events (see [35, 53]). However, the activation and proliferation of TEs may be influenced by the ability to cheat cellular silencing controls [53] and the autonomous elements containing a complete polygenic chain, regulators and both LTRs can do that [54]. The absence of some regions could make these elements non-autonomous. In the present study, more non-autonomous sequences (8-folds) were found in the three *Capsicum* datasets than potentially autonomous ones. This result suggests that these repetitive element classes may have undergone different events of degeneration along with genomes differentiation.

The putative autonomous elements recovered in the present analysis, i.e., ten sequences of Tekay/Del, two sequences of Athila/Tat of *C. annuum* and seven of CRM, varied between the datasets. This idea follows the proposal of the independent fate of TEs among genomes [55], and it can be exemplified by the occurrence of some Tekay/Del elements exclusive in *C. baccatum* compared to three others found in *C. annuum* and *C. chinense*. Also, we can mention the thirty-fold difference in CRM amount in *C. baccatum* in relation to *C. annuum* and *C. chinense*. This result is in accordance with Hawkins et al. [56] report, which suggests that in *Gossypium* species, different lineages of LTR-RTs evolved at different moments along with genome evolutionary history, generating a threefold difference in DNA content among diploid species. In another example, De Castro Nunes et al. [15] observed also a greater accumulation of CRM copies in the diploid *Coffea* species in comparison to the hybrid tetraploid *C. arabica*.

### Not all LTR-RT rich regions in *Capsicum* chromosomes are heterochromatin hotspots

It is well established in the literature that TEs, especially LTR-RT superfamilies, occupy “specific” chromosomal regions, with the consensus that *Copia* elements are distributed preferentially along the chromosomes associated with euchromatin, while *Gypsy* elements are resident in heterochromatin-rich regions (see [7]). In *Coffea*, *Brachiaria,* and *Secale*, for example, *Gypsy* probes were located in proximal heterochromatin-rich chromosome regions [17, 57, 58], but in *Gossypium* species, *Gypsy* probes were hybridized along chromosomes [59]. However, when the elements are considered according to their phylogenetic positions, i.e., lineages of *Copia* and *Gypsy* [5, 7, 60], it becomes evident that there are many differences in the TE distribution profiles, in both plants (see [10, 61]) and animals [62, 63]. Thus, it seems wiser to believe that each element has its characteristics, including chromosomal position, genome impact, epigenetic influence, diversification rate, and other features.

Previous studies using FISH in *Capsicum* spp. have been restricted to rDNA probes [25], which demonstrated that *C. baccatum* accumulates more in terminal 35S rDNA sites compared to *C. annuum* and *C. chinense*, which exhibited just two to four pairs. Moscone et al. [21, 64], Scaldaferro et al. [24] and Martins et al. [27] reports have shown wide variability in the presence of terminal, interstitial and proximal heterochromatic bands in *Capsicum* species, such as the large and minor heterochromatic terminal bands in *C. annuum*, *C. chinense,* and *C. baccatum* observed in the present study. FISH results using different LTR-RT probes showed hybridization signals accumulated from proximal (CRM) to interstitial region (Athila/Tat and Tekay/Del), scattered, or minor dots along chromosomes (Tekay/Del, Oryco and Tork), such as in *Brachiaria* [14]. However, no preferential accumulation or strong signals were found in terminal chromosome regions, suggesting that the LTR-RT families analyzed have no accumulation at regions containing rDNA or terminal heterochromatin in *Capsicum* chromosomes.

FISH using the Athila/Tat probe strongly hybridized at proximal to interstitial regions in almost all the chromosomes of *Capsicum*. There was also no evident co-location with heterochromatic regions, although there were small AT- and GC-rich bands in few chromosomes, i.e., without evident correlation with heterochromatin hotspots. In this case, the scattered signals (or dots) observed after FISH with Tekay/Del and Oryco probes are in agreement with the concept of dispersed localization of retroelements within plant genomes, but without dependence on co-localization with heterochromatin blocks. This Athila/Tat dispersion pattern, such as interstitial dots, has been described by Park et al. [65] in *C. annuum*. Using the *Passiflora edulis* for comparison, members of *Ty*3/*Gypsy* superfamily were the most accumulated, and their sequences appeared scattered along chromosomes, including at the pericentromeric regions [66]. In some Solanaceae species, such as tomato and peppers, elements of the Tekay/Del *Gypsy* superfamily had a scattered accumulation profile as reported by Park et al. [65], with hybridization in the chromosomes of *Solanum lycopersicum* (tomato) and *Capsicum annuum* (pepper), in which pepper had a higher number and more intense signals than those observed in tomato.

Different from the other LTR-RT probes, the CRM probe exhibited intense signals in the proximal regions, associated with centromeres, and in *Capsicum* chromosomes, these regions were rich in CMA^+^ and DAPI^+^ signals. The centromeric retrotransposon lineage of chromovirus (also called centromeric retrotransposon of maize or CRM) occurs preferentially in proximal chromosome regions. CRMs carry particular domains called chromodomain (CHRomatin Organization MOdifer DOMAIN) and CR motifs that have potential to interact with the CENH3 centromeric protein and to participate in the centromere function [37, 67]. FISH using CRM lineage probes have been described in several plant species, for example in some monocotyledon groups [58, 68, 69], suggesting that besides the association with specific centromeric proteins, this accumulation may also be associated with recombination-poor regions.

## Conclusions

This comparative cytogenomic analysis using the three most economically important *Capsicum* species showed great diversity in genome composition, although there is a closer approximation between *C. annuum* and *C. chinense* when compared to *C. baccatum,* as suggested by the phylogeny. The dataset screening of these three species showed that there was a differential accumulation of transposable elements, especially those from the lineages Tekay/Del, CRM and Athila/Tat from *Gypsy*, while those of the *Copia* superfamily were underrepresented. From a chromosomal point of view, these transposable elements were dispersed along the chromosomes (*Copia*) as well as in blocks (*Gypsy*), highlighting those of the CRM lineage that predominated the centromeric region. Another aspect is that LTR-RTs are not always associated with heterochromatin-rich regions. These data support the idea of the independent fate of LTR-RTs. Such genomic and chromosomal differences between closely related species should be taken into account in breeding programs, as they may interfere with the success of interspecific crosses and the introgression of agronomic traits of interest. *Capsicum* spp. proved to be a good model for most studies on the repetitive fraction, from both genomic and chromosomal points of view, considering the diversity in the accumulation and genomic distribution of LTR-RTs.

## Methods

### Plant materials

Seeds of *Capsicum annuum* cv. Criollo de Morelos (accession GBUEL145), *C. chinense* (accession GBUEL27) and *C. baccatum* (accession GBUEL118), identified by Dr. Leandro S. A. Gonçalves, were obtained from the gene bank of Londrina State University. The samples were sowed in 128-cell polystyrene trays containing the substrate Vivatto®. Ten seedlings of each species were grown in the Cytogenetics and Plant Diversity Laboratory greenhouse.

### Genomic analysis

The following three genomes used for bioinformatics analysis were obtained from Pepper Genome Platform (http://peppergenome.snu.ac.kr/): scaffolds from *Capsicum annuum* v.1.6, *C. chinense* v.1.2, and *C. baccatum* v.1.2. Files were used to search for autonomous and non-autonomous LTR-RTs. For genomic comparisons, a database was built to run a local BLAST, which contained all the TEs conserved protein sequences from REXdb [5], GypsyDB [61], RepBase [70] and NCBI (http://www.ncbi.nlm.nih.gov/), containing 283,676 protein sequences. To identify the rDNA sequences, a second database was compiled comprising nucleotides 35S and 5S rDNA sequences from different organisms, obtained from NCBI, containing 1652 sequences.

The repetitive fraction (transposable elements, 35 and 5S rDNA) evaluation was conducted by the Blast version 2.2.28+, comparing the genomic dataset against the local databases. The parameters used were E-value 10e-4, max target seqs 1 and the remaining were set by program default. The results obtained are plotted in Table S1, which includes quantitative and qualitative estimates according to the phylogeny proposal by Neumann et al. [5] as well as 35S and 5S rDNA fractions. The aim was to recognize and to differentiate retrotransposons in different lineages, and produce a more refined in situ hybridization, and to support the idea of the independent fate of these elements. The sequences were grouped into the superfamilies *Copia* (Ale, Alesia, Angela, Bianca, Bryco, Lyco, Gymco, Ikeros, Ivana, Osser, SIRE, TAR and Tork lineages) and *Gypsy* (CRM, Chlamyvir, Galadriel, Tcn1, Reina, Tekay/Del, Athila, Tat, Ogre, Retand, Phygy, and Selgy lineages). Due to their similarity, Athila, Tat, Ogre, and Retand members have been referred as Athila/Tat clade.

### Autonomous and non-autonomous estimates and primer design

LTR_STRUC [30] has been used to compare and search for LTR-retrotransposons in the reference genome of *Capsicum annuum*. Putative retrotransposons sequences were then classified into *Gypsy* and *Copia* superfamilies according to their similarity measured against the Gypsy Database protein domains (http://www.gydb.org/index.php/Main_Page) by Genewise alignment [71] and annotated with Artemis [32]. The putative elements were then used as a database for BLAST rounds against the other datasets.

The BLAST output files were organized with sequences greater than 3500 bp and more than 70% identity, and they were used to search for sequences with both LTRs with the Dotter l/b using program default parameters [31]. Subsequently, sequences that carried both LTRs were submitted to the online BLAST at NCBI to search for the presence of conserved domains. The sequences were also screened by the presence of stop codon by the Pfam online tool [72]. The complete nucleotide sequences with the correct protein order were aligned with the MAUVE [34] program and grouped as per the graphic similarity. These data were then validated by aligning them with CLUSTALW [33]. From each group, a representative sequence was chosen for the graphic annotation using the IBS [73] program. To understand the relationships among these sequences, the alignment was used to construct a bootstrapped maximum likelihood (ML) phylogenetic tree (1000 bootstraps) in MegaX [74]. The ML tree was generated with GTR (general time-reversible) mutation model, gamma-distributed and invariant sites (G + I) rates among sites, and the heuristic method was nearest-neighbor-interchange (NNI). To understand how these putative autonomous elements are dispersed and accumulated along with the datasets, HeatMapper tool [75] was used in the three datasets through two approaches, one being with a representative sequence from each group of putative autonomous elements and the other with the reverse transcriptase region of these groups.

The most conserved stretch of the reverse transcriptase of each LTR-RT lineage was used for primer designing with the custom primers, OligoPerfect TM Designer tool of Thermo Fisher Scientific (http://tools.thermofisher.com, see Table 2). The primers’ viability was assessed with the PCR primer stats tool (http://www.bioinformatics.org).

**Table 2:** List of primers of reverse transcriptase sequences, that have been designed for probes obtaining and FISH

Elements	Primers
Athila/Tat - RT	F 5′ GGGTGGTATTGCTTCTTGGA 3′ R 5′ GAATCACCTACCACAGAG 3′
CRM - RT	F 5′ CCACCAACAAGATAACGG 3′ R 5′ CCATCCATTCATAGAGACC 3′
Tekay/Del - RT	F 5′ GTTCAGGGTGCCAAGTGT 3′ R 5’GGGCGTTAGTCAACCTGAAG 3′
Ivana/Oryco - RT	F 5′ GGTTCAAGGATCGGTTGATAG 3′ R 5′ GTTGAGCTTGCACACCATGT3’

*RT* Reverse transcriptase

### DNA extraction, PCR and LTR-RT probes

DNA was isolated from young leaves of each species using the cetyltrimethylammonium bromide (CTAB) method [76], purified with phenol:chloroform (1:1, v/v), chloroform:isoamyl alcohol (24:1, v/v) and RNase (1 mg mL^− 1^) and precipitated in 100% absolute ethanol. The samples were eluted in 10 mM Tris–HCl pH 8 and the concentrations were estimated using a NanoDrop 2000 Spectrophotometer (Thermo Scientific).

The LTR-RT probes were obtained by PCR using specific primers for each lineage, with *C. annuum* as a DNA template. A standard PCR [5 U μL^− 1^ *Taq* polymerase (0.5 μL), 10× buffer (2.5 μL), 50 mM MgCl_2_ (1.5 μL), 10 mM dNTP (1 μL), 5 mM primers (2 μL each) and H_2_O up to a final volume of 25 μL] was used under the following conditions: 94 °C for 2 min, 30 cycles of 94 °C for 40 s, 59 °C for 40 s and 72 °C for 1 min, and a final extension of 72 °C for 10 min. The reactions were tested via electrophoresis in an agarose gel at 3 V cm^− 1^ and stained with ethidium bromide. The probes for each LTR-RT lineage were obtained through the re-amplification of PCR products that involved labeling with biotin-11-dUTP (*Gypsy* families) or Cy3-dUTP (*Copia* families).

### Cytogenetic analysis

The root tips were pretreated with 0.5% colchicine (1 h 30 min) and fixed in ethanol-acetic acid (3:1, v:v). The fixed material was treated in a solution of 2% cellulase and 20% pectinase and squashed in a drop of 60% acetic acid. After liquid nitrogen freezing, the coverslips were removed, and the slides were air-dried.

For fluorescence in situ hybridization, slides received a mix containing a solution (30 μL) composed of 100% formamide (15 μL), 50% polyethylene glycol (6 μL), 20× SSC (3 μL), 100 ng of calf thymus DNA (1 μL), 10% SDS (1 μL) and 100 ng of probes (4 μL). The mix was denatured at 90 °C for 10 min, and hybridization was performed at 37 °C for 24 h in a humid chamber. Post-hybridization washes were carried out with 70% stringency, using an SSC buffer, with pH 7.0. After the probe detection with an avidin-fluorescein isothiocyanate (FITC) conjugate, washes were performed in 4× SSC/0.2% Tween-20 at room temperature. The slides were mounted with 25 μL of DABCO, a solution composed of glycerol (90%), 1,4-diaza-bicyclo (2.2.2)-octane (2.3%), 20 mM Tris–HCl, pH 8.0 (2%), 2.5 mM MgCl_2_ (4%) and distilled water (1.7%) in addition to 1 μL of 2 μg mL^− 1^ 4,6′-diamidino-2-phenylindole (DAPI).

For C-CMA/DAPI banding, the samples were incubated in a sequence of 45% acetic acid (8 min), saturated solution of Ba(OH)_2_ at room temperature (8 min) and in 2× saline sodium citrate at 60 °C (1 h and 30 min). After this, the slides were stained with a CMA_3_ for 90 min and DAPI for 30 min [77] and mounted in a solution of McIlvaine buffer plus glycerol (1:1, v:v) with 2.5 mM MgCl_2_.

The chromosome images were acquired in greyscale with a Leica DM4500 B microscope coupled with a DFC300FX camera, pseudo-colored (blue for DAPI, greenish-yellow for FITC and red for Cy3) and contrasted using GIMP 2.8 Linux.

## Supplementary information

**Additional file 1: Figure S1.** Mauve alignment of Tekay/Del sequences from *Capsicum* genomes. **Figure S2.** Mauve alignment of CRM sequences from *Capsicum* genomes. **Figure S3.** Mauve alignment of Athila sequences from *Capsicum* genomes. **Figure S4.** FISH using LTR-RTs probes against metaphases and prometaphases of *Capsicum* species. **Figure S5.** FISH using LTR-RTs probes against metaphases and prometaphases of *Capsicum* species. **Figure S6.** FISH using LTR-RTs probes against metaphases and prometaphases of *Capsicum* species. **Table S1.** Frequency and relative values of repetitive fraction in the three *Capsicum* genomes. **Figure S7.** Phylogeny for *Capsicum* adapted from Carizzo-Garcia et al. [45]

## Data Availability

Original sequences of these three genomes can be accessed at Pepper Genome Platform (http://peppergenome.snu.ac.kr/).

## Abbreviations

CRM: Centromeric Retrotransposon of Maize
ERV: Endogenous Retrovirus
FISH: Fluorescence in situ hybridization
LINEs: Long Interspersed Elements
LTR-RT: LTR-retrotransposons
LTRs: Long Terminal Repeats
pg: Picograms
rDNA: Ribosomal DNA
SDS: Sodium Dodecyl Sulfate
SINEs: Short interspersed nuclear elements
SSC: Saline-Sodium Citrate
TEs: Transposable elements
