# Supplementary material for: Genome relationships and LTR-retrotransposon diversity in three cultivated Capsicum L. (Solanaceae) species
Source: BMC Genomics. 2020 Mar 17;21:237. doi: 10.1186/s12864-020-6618-9 (PMC7076952; doi:10.1186/s12864-020-6618-9)
Supplement: Supplementary file 1 — Additional file 1: Figure S1. Mauve alignment of Tekay/Del sequences from Capsicum genomes. Figure S2. Mauve alignment of CRM sequences from Capsicum genomes. Figure S3. Mauve alignment of Athila sequences from Capsicum genomes. Figure S4. FISH using LTR-RTs probes against metaphases and prometaphases of Capsicum species. Figure S5. FISH using LTR-RTs probes against metaphases and prometaphases of Capsicum species. Figure S6. FISH using LTR-RTs probes against metaphases and prometaphases of Capsicum species. Table S1. Frequency and relative values of repetitive fraction in the three Capsicum genomes. Figure S7. Phylogeny for Capsicum adapted from Carizzo-Garcia et al. [45] [file 12864_2020_6618_MOESM1_ESM.pdf]

Supplementary Materials

Genome relationships and LTR retrotransposon diversity in three cultivated *Capsicum* L. (Solanaceae) species. Assis R, Baba VY, Cintra LA, Gonçalves LSA, Rodrigues R, Vanzela ALL.

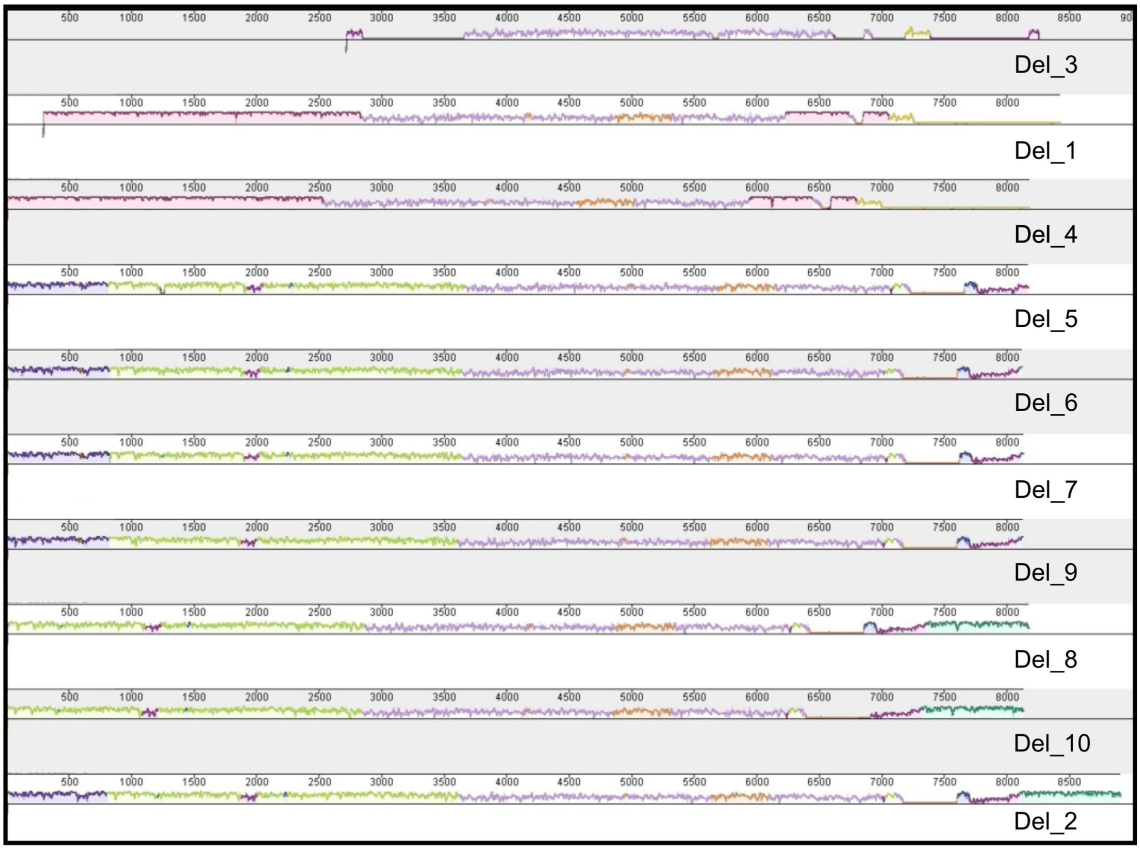

Supplementary figure 1. **Mauve alignment of Tekay/Del sequences from *Capsicum* genomes.** The graphical similarity was used as a parameter to determine the clusters of the Del sequences. Del_3 from *C. annuum* make a group. Del_1 from *C. annuum* and Del_4 from *C. chinense* were grouped together, forming the second group. The third group was formed by sequences Del_5, Del_6, Del_7 and Del_9, exclusively from *C. baccatum*, such as the group formed by Del_8 and Del_10. The sequence Del_2 from *C. annuum* was grouped alone. Note the differences among these sequences by the length and position of LTRs.

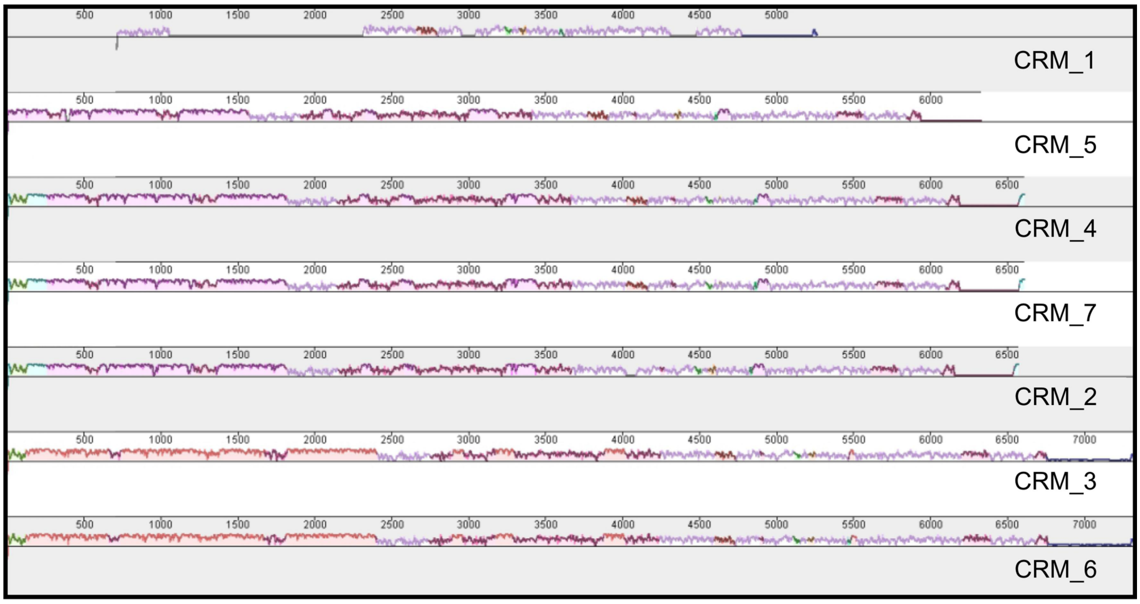

Supplementary figure 2. Mauve alignment of CRM sequences from *Capsicum* genomes. The graphical similarity was used as a parameter to determine the clusters of the CRM sequences. CRM_1 from *C. annuum* and CRM_5 from *C. baccatum* made each one a group. The sequences CRM_4 from *C. annuum*, CRM_7 from *C. chinense* and CRM_2 from *C. annuum* were grouped together and the last group was formed by the sequences CRM_3 from *C. annuum* and CRM_6 from *C. chinense*. Note the differences among these sequences by the length and position of LTRs.

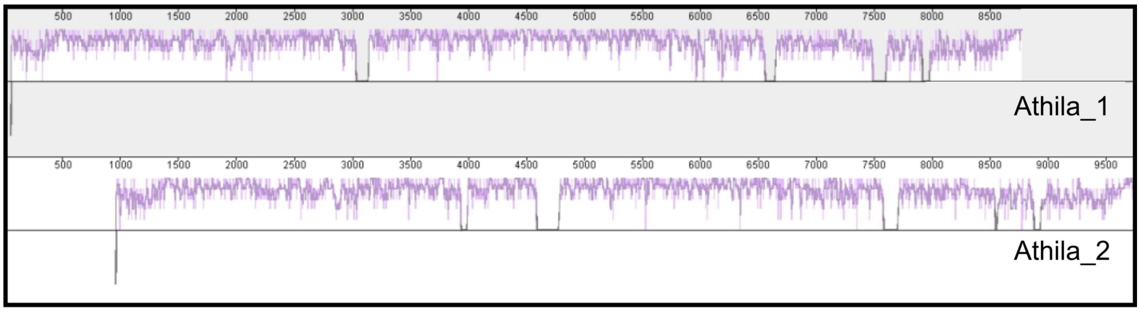

Supplementary figure 3. Mauve alignment of Athila sequences from *Capsicum* genomes. The graphical similarity was used as a parameter to determine the clusters of the Athila sequences from *C. annuum*. Each sequence made a group, note the differences between these sequences, especially for the length.

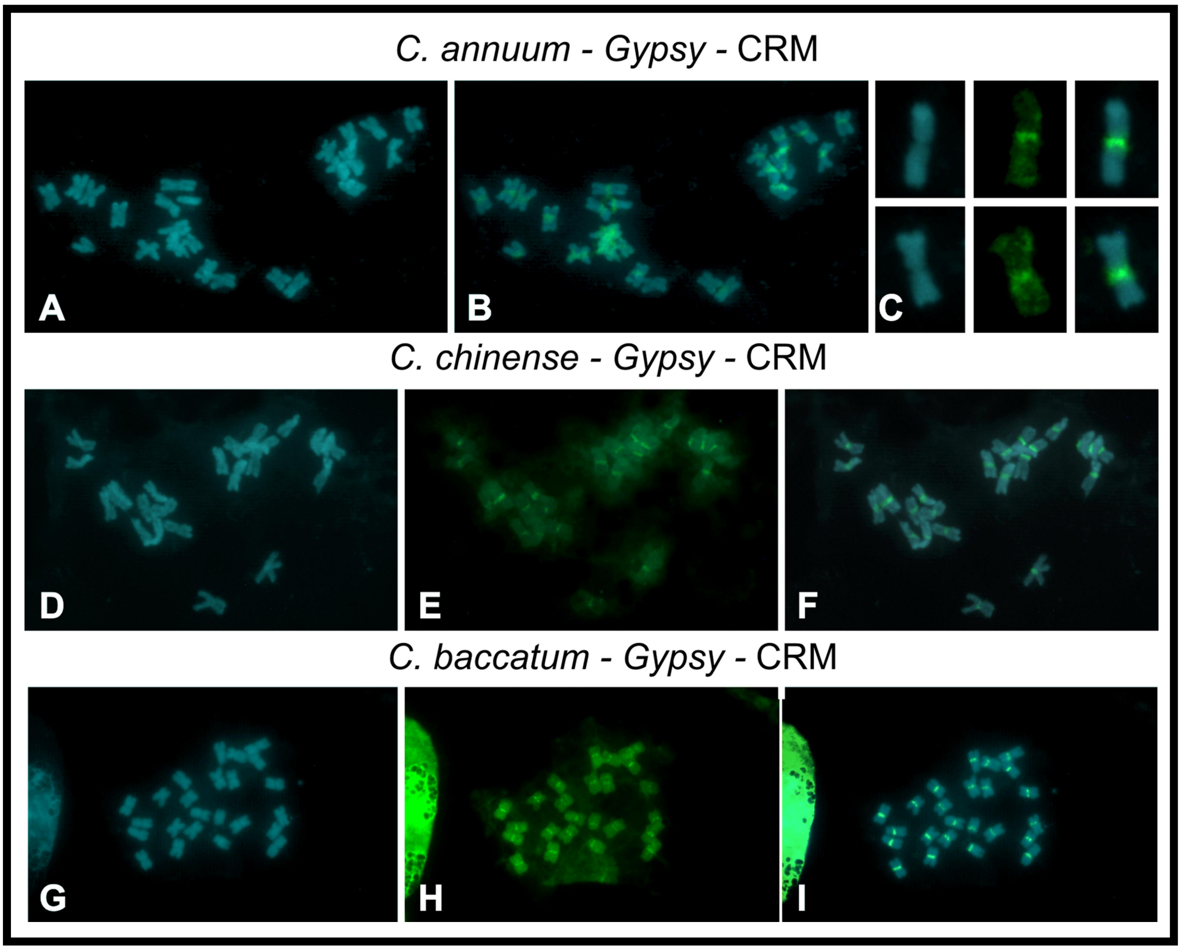

Supplementary figure 4. FISH using LTR-RTs probes against metaphases and prometaphases of *Capsicum* species. Chromosomes were counter-stained with DAPI (blue), CRM probes labelled with biotin-11-dUTP / avidin–FITC conjugate (green). The CRM probe exhibited a proximal pattern of hybridization signals in all species. **(A - B)** *C. annuum*. **(C)** Highlighted chromosomes from *C. annuum* exhibiting the proximal signal from CRM probe have been obtained from a different metaphase. **(D - F)** *C. chinense.* **(G - I)** *C. baccatum*. Note that the signals in *C. baccatum* were more intense than the ones observed in *C. annuum* and *C. chinense*.

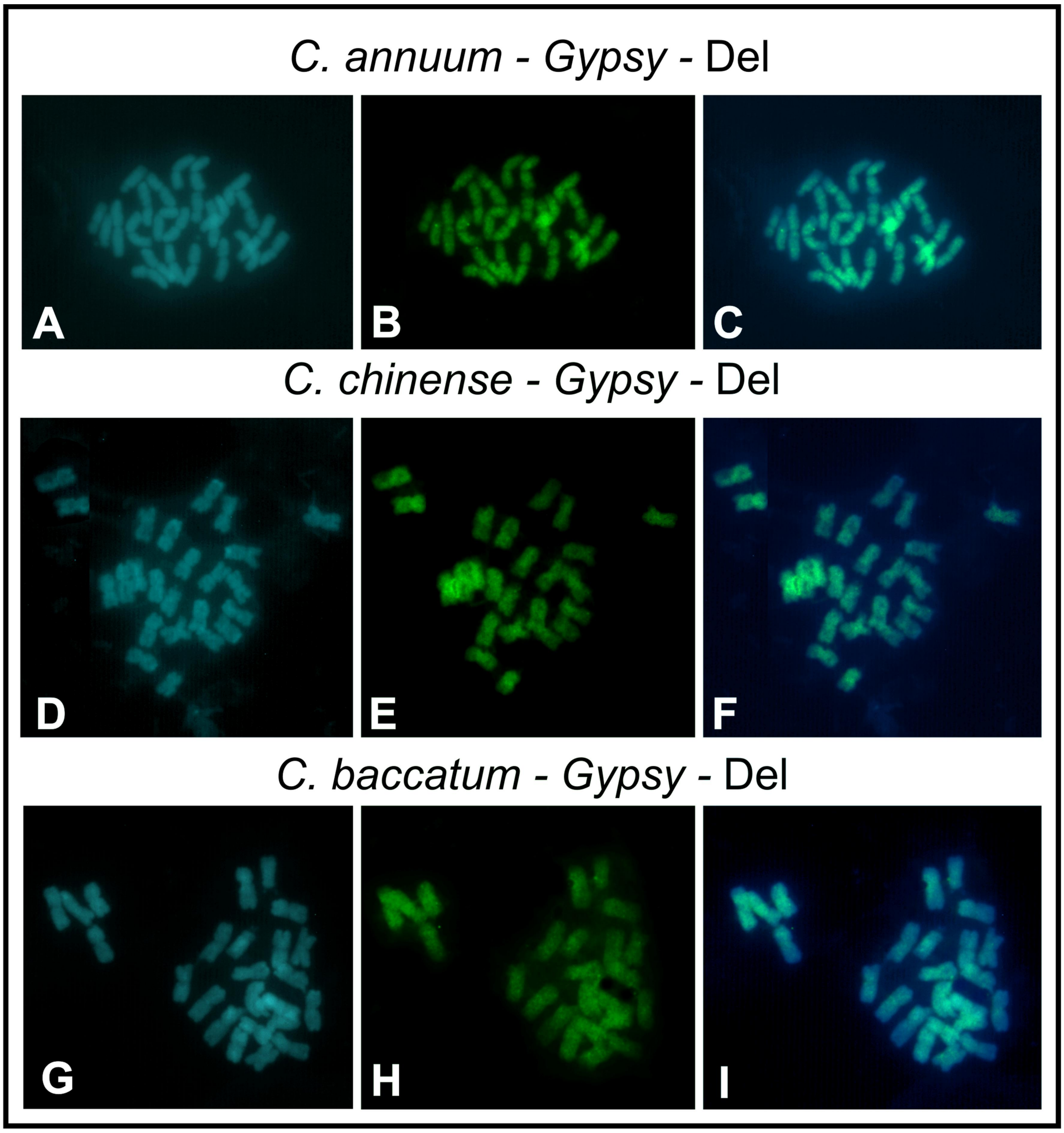

Supplementary figure 5. FISH using LTR-RTs probes against metaphases and prometaphases of *Capsicum* species. Chromosomes were counter-stained with DAPI (blue), Tekay/Del probes labelled with biotin-11-dUTP / avidin–FITC conjugate (green). The *Gypsy* Tekay/Del probe exhibited greater accumulation signals in *C. annuum* **(A-C)** than in the other two species, followed by *C. chinense* **(D-F)** than by *C. baccatum* **(G-I)**, which showed less signals, although some chromosomes exhibit more intense signal than the others.

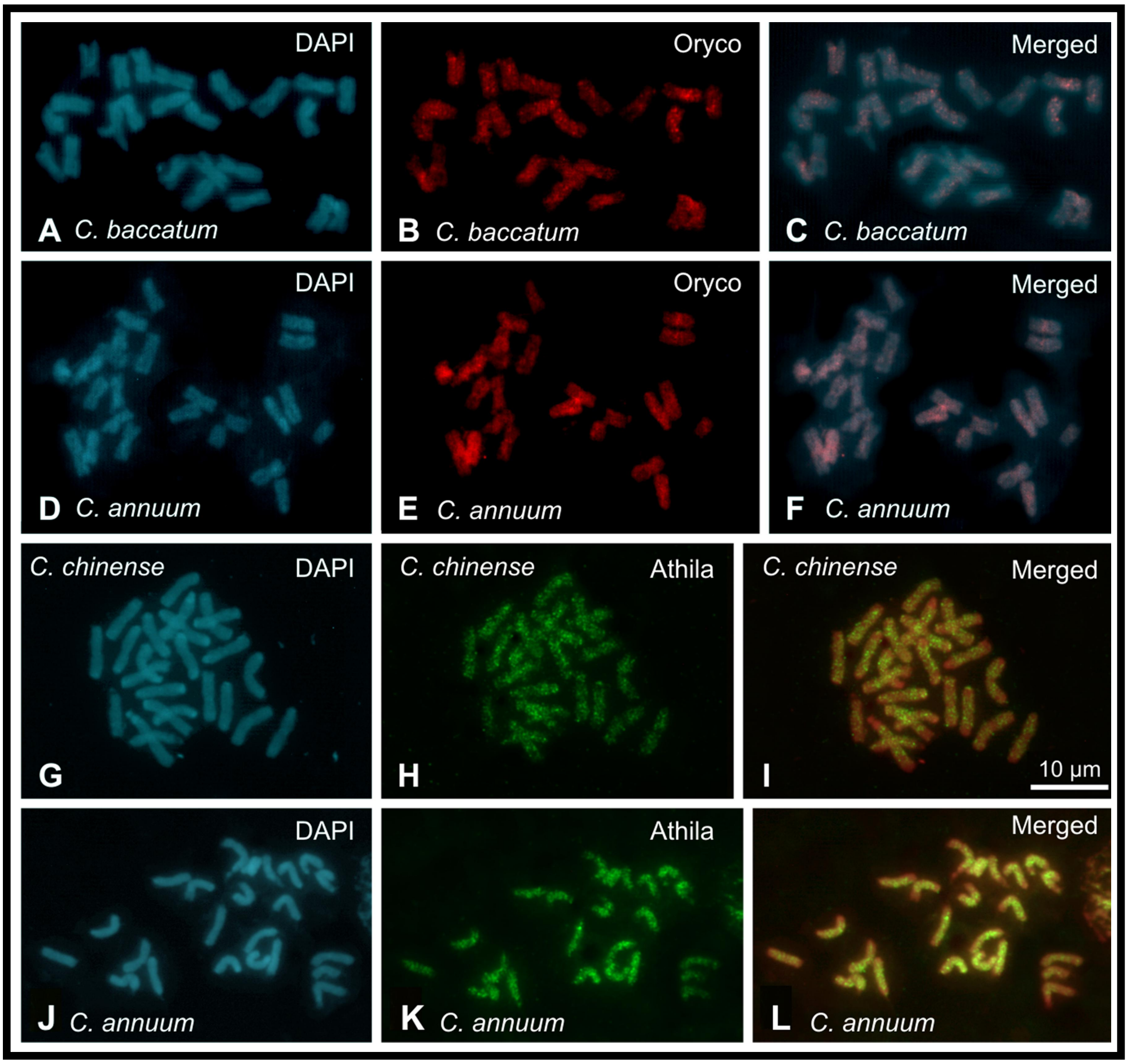

Supplementary figure 6. FISH using LTR-RTs probes against metaphases and prometaphases of *Capsicum* species. Chromosomes were counter-stained with DAPI (blue), *Copia* probes with Cy3-11-dUTP (red) and *Gypsy* probes labelled with biotin-11-dUTP / avidin–FITC conjugate (green). The *Copia* Ivana/Oryco exhibited few hybridization signals scattered along chromosomes, with a low accumulated profile in both *C. baccatum* **(A – C)** and *C. annuum* **(D - F)**. The *Gypsy* Athila/Tat probe showed accumulation in the pericentromeric to interstitial regions of all chromosomes of *C. chinense* **(G - I)** and *C. annuum* **(J – L)**, although some chromosomes exhibit a greater accumulation than others, but this was restricted a just few chromosomes pairs. **(I)** Bar represents 10 µm.

Supplementary Table 1. Frequency and relative values of repetitive fraction in the three *Capsicum* genomes.

Species/Lineages		*C. annuum*				*C. chinense*				*C. baccatum*			
		num seq	NR (%)	size bp	size CR%	num seq	NR (%)	size bp	size CR%	num seq	NR (%)	size bp	size CR%
Copia	Ivana/Oryco	0	0.00	0	0.00	0	0.00	0	0.00	0	0.00	0	0.00
	Sire	6065	1.38	1161677	1.38	7037	1.75	1198669	0.23	2386	0.64	403471	0.51
	Ale/Retrofit	1292	0.29	175175	0.21	1959	0.49	156214	0.03	996	0.27	139797	0.18
	Alesia	4	0.00	139	0.00	14	0.00	470	0.00	3	0.00	103	0.00
	Bryco	12	0.00	476	0.00	34	0.01	1236	0.00	4	0.00	116	0.00
	Gymco	22	0.01	804	0.00	85	0.02	3233	0.00	50	0.01	2101	0.00
	Osser	45	0.01	1335	0.00	65	0.02	2534	0.00	6	0.00	241	0.00
	Ikeros	0	0.00	0	0.00	0	0.00	0	0.00	0	0.00	0	0.00
	Lyco	7	0.00	341	0.00	12	0.00	499	0.00	17	0.00	610	0.00
	Angela	7	0.00	357	0.00	18	0.00	726	0.00	0	0.00	0	0.00
	Tar	2928	0.67	1206559	1.43	66	0.02	2975	0.00	6550	1.77	4755842	5.98
	Bianca	12	0.00	524	0.00	17	0.00	551	0.00	7	0.00	280	0.00
	Tork	2452	0.56	439027	0.52	2312	0.58	354600	0.07	2223	0.60	475825	0.60
Total Copia		12846	2.92	2986414	3.54	11619	2.90	1721707	0.33	12242	3.31	5778386	7.26
Gypsy	Tekay/Del	314401	71.57	57116162	67.68	269454	67.17	492211359	95.43	63219	17.08	12491685	15.70
	Reina	1340	0.31	174430	0.21	1757	0.44	157406	0.03	1012	0.27	142756	0.18
	CRM	4645	1.06	942613	1.12	3834	0.96	640754	0.12	12925	3.49	2867265	3.60
	Galadriel	1540	0.35	346734	0.41	3448	0.86	692338	0.13	1595	0.43	429635	0.54
	Tcn1	6	0.00	275	0.00	26	0.01	990	0.00	6	0.00	266	0.00
	Chlamyvir	0	0.00	0	0.00	0	0.00	0	0.00	0	0.00	0	0.00
	Selgy	9	0.00	771	0.00	15	0.00	482	0.00	5	0.00	211	0.00
	Phygy	6	0.00	164	0.00	22	0.01	760	0.00	8	0.00	759	0.00
	OTA/Retand	0	0.00	0	0.00	0	0.00	0	0.00	0	0.00	0	0.00
	OTA/Ogre	34382	7.83	6697714	7.94	37020	9.23	6980112	1.35	112366	30.36	28130817	35.36
	OTA/Tat	23038	5.24	4904332	5.81	21420	5.34	4370374	0.85	24526	6.63	5867540	7.38
	OTA/Athila	12740	2.90	2205937	2.61	16090	4.01	2674968	0.52	89251	24.12	403471	0.51
Total Gypsy		392107	89.26	72389132	85.77	353086	88.02	507729543	98.43	304913	82.40	50334405	63.27
Bel		257	0.06	10350	0.01	871	0.22	43039	0.01	225	0.06	9014	0.01
DIRS		12575	2.86	5060780	6.00	0	0.00	0	0.00	3619	0.98	1430071	1.80
Penelope		4	0.00	138	0.00	15	0.00	1266	0.00	1	0.00	22	0.00
Total LTR-RTs		405214	92.24	75386034	89.32	365591	91.14	509495555	98.78	317381	85.77	56121827	70.55

Species/Lineages		*C. annuum*				*C. chinense*				*C. baccatum*			
		num seq	NR (%)	size bp	size CR%	num seq	NR (%)	size bp	size CR%	num seq	NR (%)	size bp	size CR%
LINE	L1	2305	2,63	246443	0.29	1958	0.49	152163	0.03	627	0.17	55746	0.07
	RTE	1010	1,15	1537280	1.82	1034	0.26	133630	0.03	2537	0.69	1052374	1.32
SINE		504	0,58	82281	0.10	313	0.08	11064	0.00	484	0.13	92928	0.12
Total Non-LTR-RTs		3819	4,36	1866004	2.21	3305	0.82	296857	0.06	3648	0.99	1201048	1.51
Total Class I		405214	92.24	77252038	91.54	368896	91.96	509792412	98.83	321029	86.75	57322875	72.06
Virus-like	Caulimovirus	499	0.11	39112	0.05	510	0.13	26444	0.01	272	0.07	23533	0.03
	Soymovirus	181	0.04	7370	0.01	279	0.07	11751	0.00	52	0.01	2239	0.00
	Cavemovirus	63	0.01	4338	0.01	56	0.01	2539	0.00	21	0.01	1095	0.00
	Tungrovirus	1	0.00	24	0.00	6	0.00	221	0.00	0	0.00	0	0.00
	Badnavirus	378	0.09	30295	0.04	463	0.12	21442	0.00	287	0.08	16230	0.02
	Pararetrovirus	249	0.06	46075	0.05	78	0.02	8670	0.00	539	0.15	142402	0.18
Total Virus-like		1371	0.31	127214	0.15	1392	0.35	71067	0.01	1171	0.32	185499	0.23
Transposons	CACTA	498	0.11	32067	0.04	969	0.24	49043	0.01	405	0.11	26019	0.03
	Merlin	1	0.00	14	0.00	6	0.00	309	0.00	0	0.00	0	0.00
	Sola	24545	5.59	4856624	5.75	23037	5.74	4843863	0.94	25145	6.79	5816849	7.31
	PiggyBac	1	0.00	71	0.00	9	0.00	662	0.00	5	0.00	275	0.00
	P	9	0.00	314	0.00	30	0.01	1882	0.00	15	0.00	627	0.00
	Novosib	3	0.00	69	0.00	13	0.00	418	0.00	0	0.00	0	0.00
	Kolobok	5	0.00	137	0.00	18	0.00	712	0.00	12	0.00	527	0.00
	hAT	3948	0.90	840183	1.00	1223	0.30	89327	0.02	2934	0.79	1234232	1.55
	PIF-Harbinger	314	0.07	11235	0.01	503	0.13	18601	0.00	254	0.07	10921	0.01
	Tc1-Mariner	88	0.02	3775	0.00	213	0.05	9885	0.00	48	0.01	2272	0.00
	MuDR	580	0.13	41665	0.05	1096	0.27	52668	0.01	416	0.11	31131	0.04
	MITEs	289	0.07	22601	0.03	486	0.12	14744	0.00	153	0.04	20893	0.03
Crypton		37	0.01	1751	0.00	95	0.02	3358	0.00	19	0.01	660	0.00
Helitrons		413	0.09	29682	0.04	904	0.23	49385	0.01	171	0.05	9904	0.01
Polintons		7	0.00	247	0.00	25	0.01	1016	0.00	5	0.00	179	0.00
Total Class II		30738	7.00	5840435	6.92	28627	7.14	5135873	1.00	29582	7.99	7154489	8.99
5S rDNA		717	0.16	89820	0.11	1370	0.34	172715	0.03	199	0.05	30499	0.04
35S rDNA		1267	0.29	1086206	1.29	849	0.21	637555	0.12	18075	4.88	14858366	18.68

Species/Lineages	*C. annuum*				*C. chinense*				*C. baccatum*			
	num seq	NR (%)	size bp	size CR%	num seq	NR (%)	size bp	size CR%	num seq	NR (%)	size bp	size CR%
Total rDNA	1984	0.45	1176026	1.39	2219	0.55	810270	0.16	18274	4.94	14888865	18.72
TOTAL	439307	100.00	84395713	100.00	401134	100.00	515809622	100.00	370056	100.00	79551728	100.00
input contigs	37989		3063651248		51917		3009228238		25349		3215433722	
mapped contigs	26372		84395713		36647		515809622		19808		79551728	
% mapped	69.42		2.75		70.59		17.14		78.14		2.47	

Repetitive fraction comparison in *Capsicum* genomes. num seq – number of sequences found after the Blast rounds. NR (%) – relative value. Size bp – total length of a repetitive class. Size CR (%) – percentage that the class represents in the scaffolds.

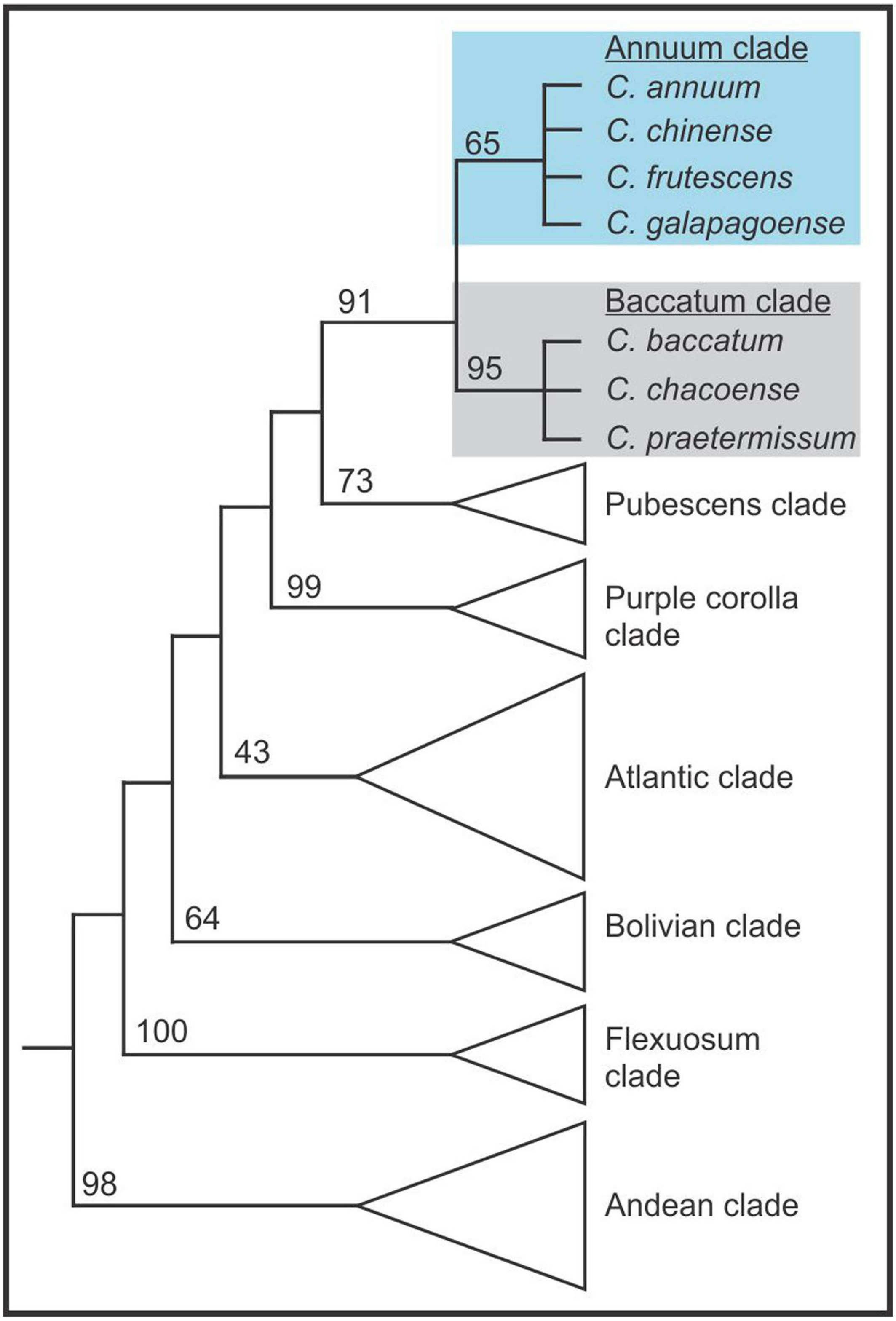

Supplementary figure 7. Phylogeny for *Capsicum* adapted from Carizzo-Garcia et al. (2016). Box in blue color highlitgh the Annuum clade, that contains *C. annuum*, *C. chinense*, *C. frutescens* and *C. galapagoense*. In gray the Baccatum clade highlitghing *C. baccatum*, *C. chacoense* and *C. praetermissum*. The support values for the clusters are the same as those found in the tree constructed by maximum parsimony analysis of Carizzo-Garcia and colleagues.
